# Supplementary material for: Local flux coordination and global gene expression regulation in metabolic modeling
Source: Nat Commun. 2023 Sep 14;14:5700. doi: 10.1038/s41467-023-41392-6 (PMC10502109; doi:10.1038/s41467-023-41392-6)
Supplement: Supplementary file 1 — Supplementary Information [file 41467_2023_41392_MOESM1_ESM.pdf]

Supplementary Information

Local flux coordination and global gene expression regulation in metabolic modeling

Gaoyang Li[1,2], Li Liu[3], Wei Du[1*] and Huansheng Cao[3*]

[1]Key Laboratory of Symbol Computation and Knowledge Engineering of the Ministry of Education, College of Computer Science and Technology, Jilin University, Changchun, 130012, China

[2]Translational Medical Center for Stem Cell Therapy and Institute for Regenerative Medicine, Shanghai East Hospital, Bioinformatics Department, School of Life Sciences and Technology, Tongji University, Shanghai, China

[3]Division of Natural and Applied Sciences, Duke Kunshan University, Kunshan, 215316, China

* Corresponding authors

Gaoyang Li: lgyzngc@tongji.edu.cn

Li Liu: li.liu@duke.edu

Wei Du: weidu@jlu.edu.cn

Huansheng Cao: hc284@duke.edu

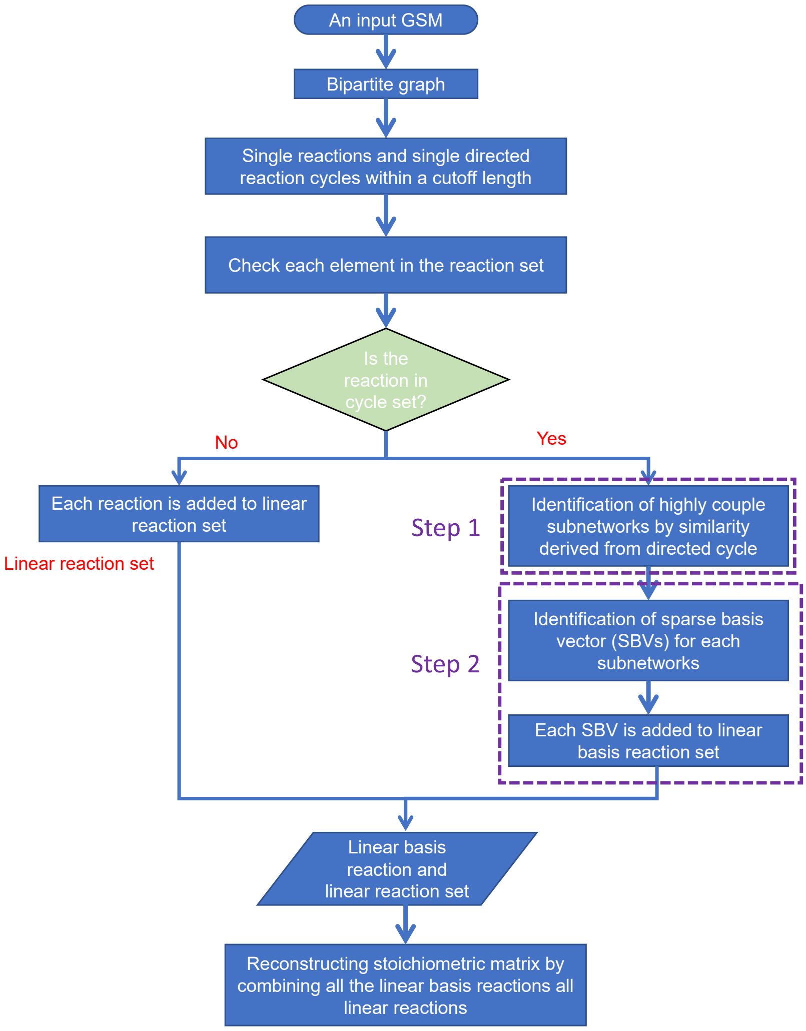

Supplementary Fig. 1 The local regulation workflow of Decrem.

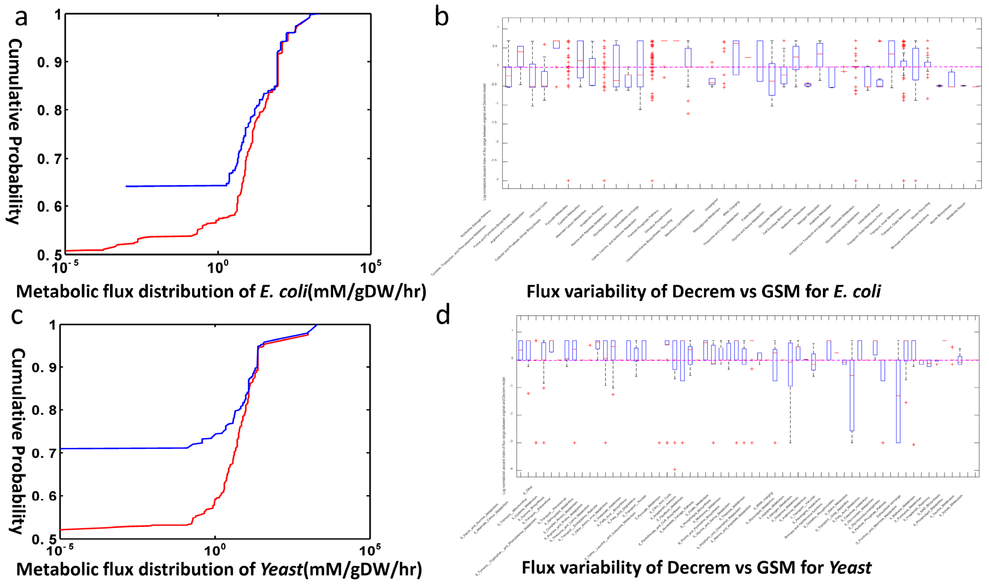

Supplementary Fig. 2 The cumulative flux distribution and flux variability. a,**c** The cumulative flux distribution of *E. coli i*AF1260 and Yeast *i*MM904 model (red) and reconstructed decoupled Decrem model (blue) by the flux variability analysis. Source data are provided as a Source Data file. **b** Comparison of pathway-specific flux variability of Decrem vs original GSM for E. coli iAF1260 (n=2711 in total, reaction number of each pathway for each sub-boxplot can be found in Source Data Fig. s2b). **d** Comparison of pathway-specific flux variability of Decrem vs original GSM for Yeast iMM904 (n=1577 in total, reaction number of each pathway for each sub-boxplot can be found in Source Data Fig. s2d). In **b** and **d**, the red center line denotes the median value (50th percentile), while the blue box contains the 25th to 75th percentiles of the dataset. The black whiskers mark the 5th and 95th percentiles, and values beyond these upper and lower bounds are considered outliers, marked with red crosses. Source data are provided as a Source Data file.

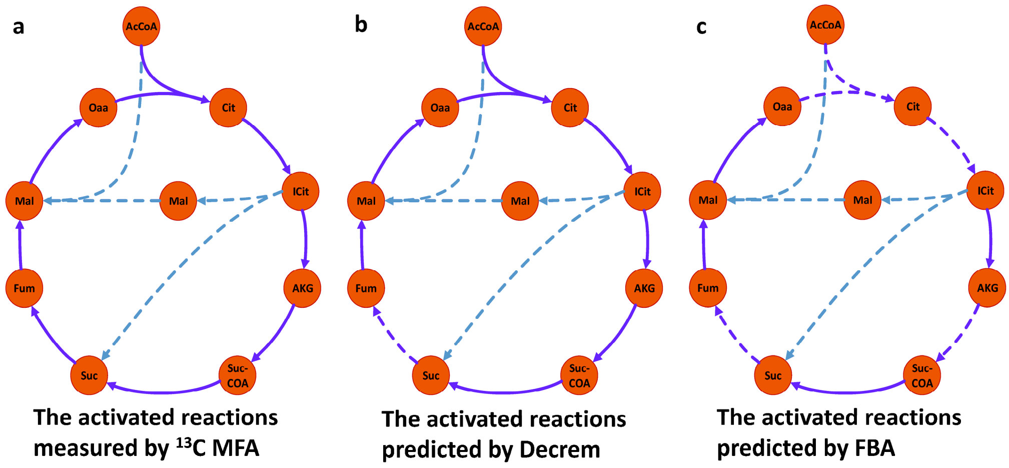

Supplementary Fig. 3 Comparison of activated reactions of TCA cycle among ^{13}C. a MFA, **b** Decrem and **c** FBA. Solid lines indicate the activated reactions and dash lines indicate the reactions having zero fluxes.

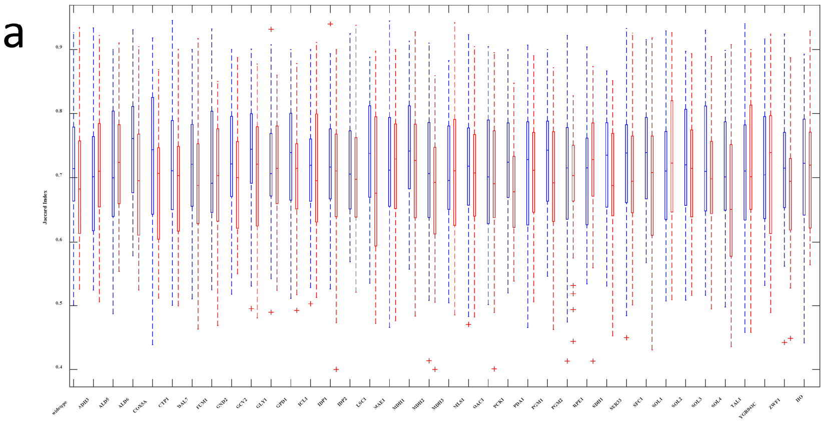

Yeast *i*MM904 model in 38 mutant strains

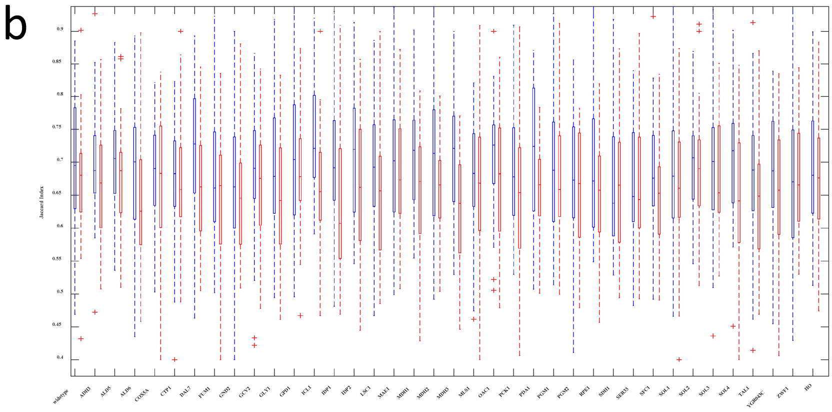

Yeast *i*DN750 model in 38 mutant strains

Supplementary Fig. 4 Jaccard index metric distribution. **a-b**, Jaccard index metric distribution of predicted fluxes by Decrem vs. the ^{13}C-MFA (blue boxplot), as well as the predictions with original GSM vs the ^{13}C-MFA (red boxplot) for *Yeast i*MM904 (**a**) and *i*DN750 (**b**) model in 38 mutant strains. (n=39 for each subplot, paired two-tailed t-test, $p<0.01$). The blue and red center line denotes the median value (50th percentile), while the blue and red box contains the 25th to 75th percentiles of the dataset. The blue and red whiskers mark the 5th and 95th percentiles, and values beyond these upper and lower bounds are considered outliers, marked with blue and red crosses. Source data are provided as a Source Data file.

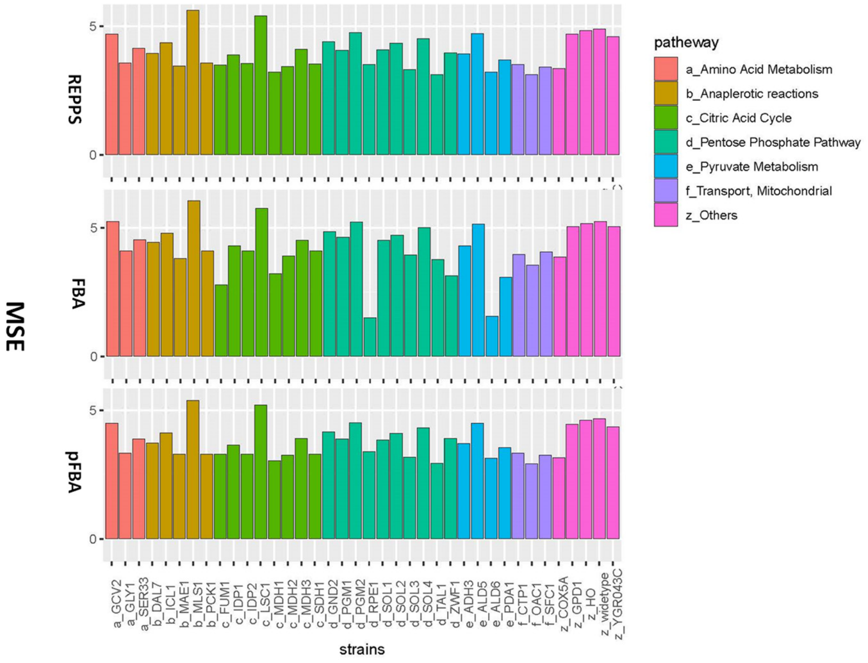

Supplementary Fig. 5 MSE distribution of predicted fluxes. MSE distribution of predicted fluxes by FBA, REPPS, and pFBA methods through the pathways where mutant genes occur. Source data are provided as a Source Data file.

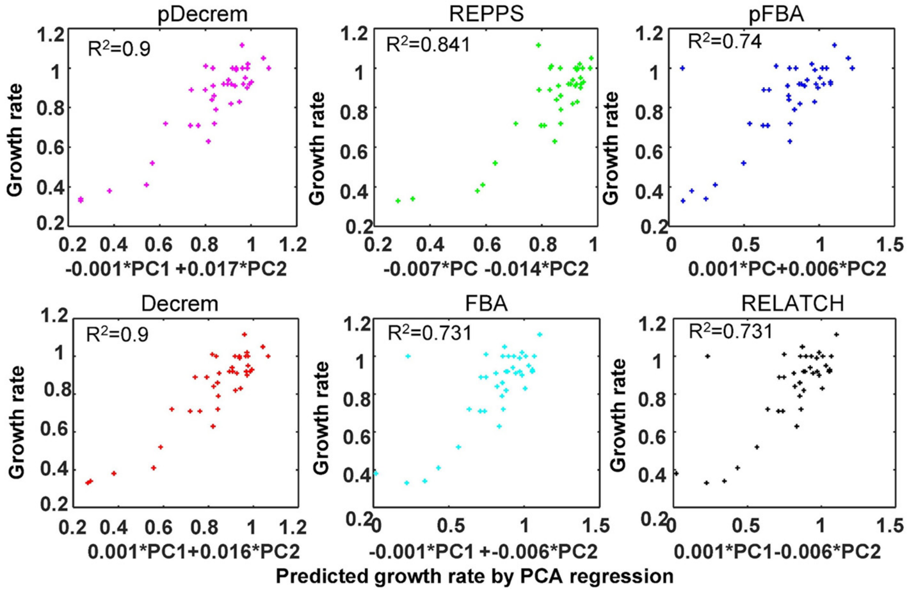

Supplementary Fig. 6 Representation of the experimentally measured and predicted growth rates. Representation of the experimentally measured (y-axis) and predicted (x-axis) growth rates with PCA regression of the top two PCs from the six test methods. Source data are provided as a Source Data file.

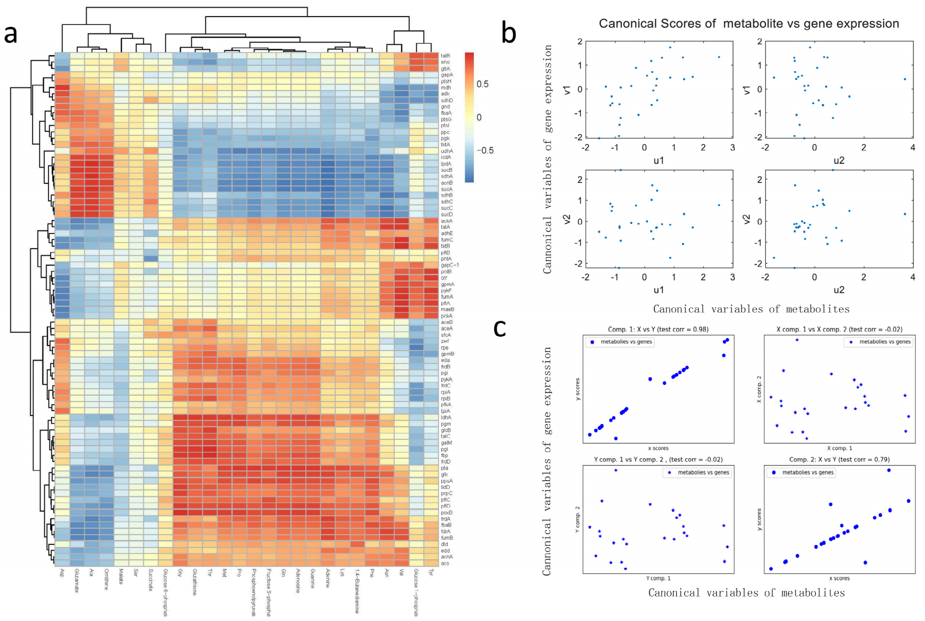

Supplementary Fig. 7 The correlation between gene expression and metabolites. a The correlation heatmap of gene expression and metabolite concentration in our experimental dataset validates the identified biomass constituent metabolites with high positive correlations with the PPP pathway and pyruvate metabolism and negative correlations with the TCA cycle. **b** The canonical correlation analysis between 85 gene expressions and all 45 candidate regulator metabolites on the Ishii et al. dataset [1]. **c** Canonical correlation analysis between the biomass metabolites and identified 32 genes in central metabolism on our experimental dataset. Source data are provided as a Source Data file.

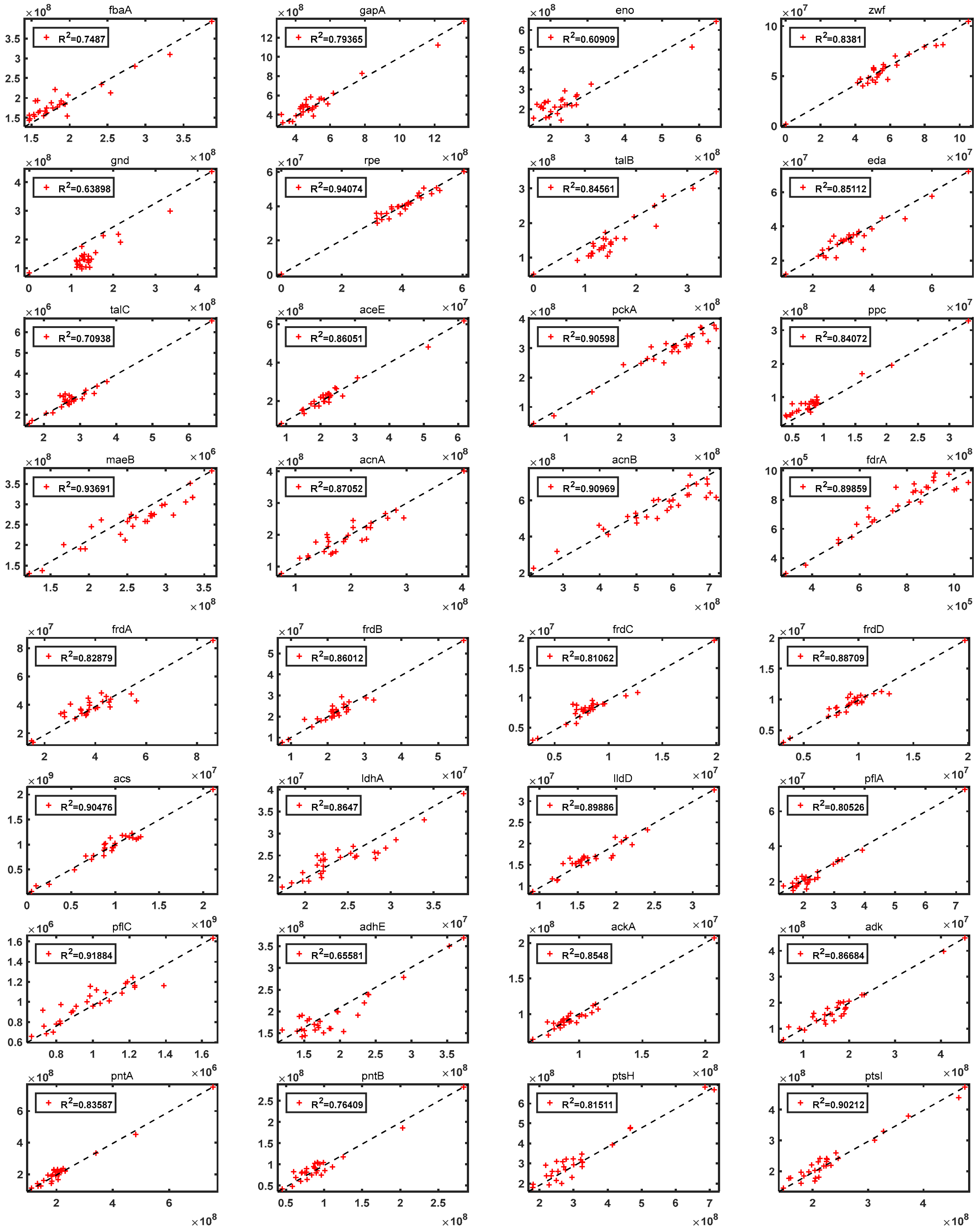

Supplementary Fig. 8 The consistency between measured and predicted expression levels. The consistency of 32 genes between measured and predicted expression levels by the PLSR methods. Source data are provided as a Source Data file.

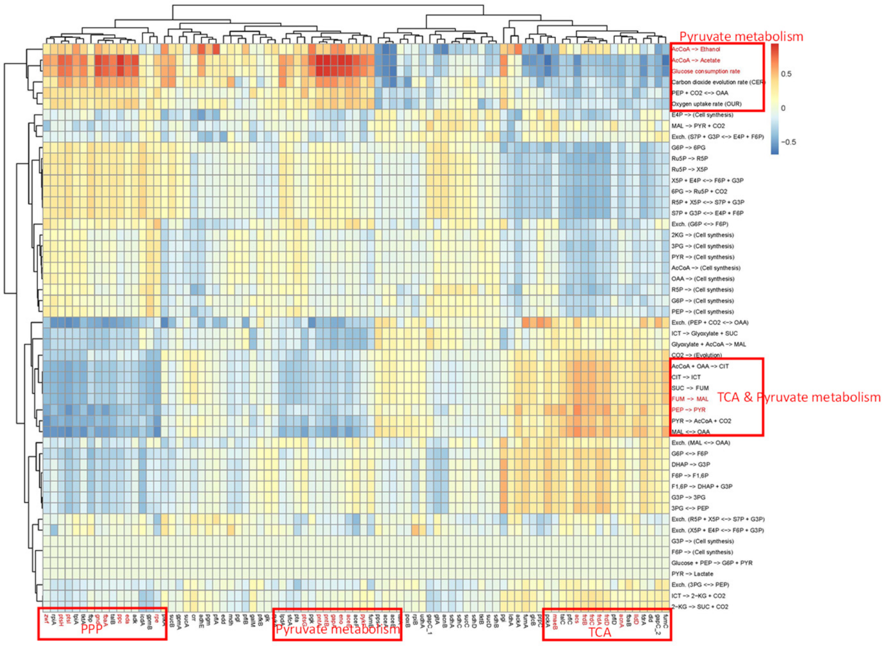

Supplementary Fig. 9 Correlation heatmap of gene expression and ^{13}C MFA flux of *E. coli* central metabolism in 24 mutant strains. Note the red-colored reactions in pyruvate metabolism are highly correlated with the three globally regulated gene clusters, indicating those reactions are growth state-specific. Source data are provided as a Source Data file.

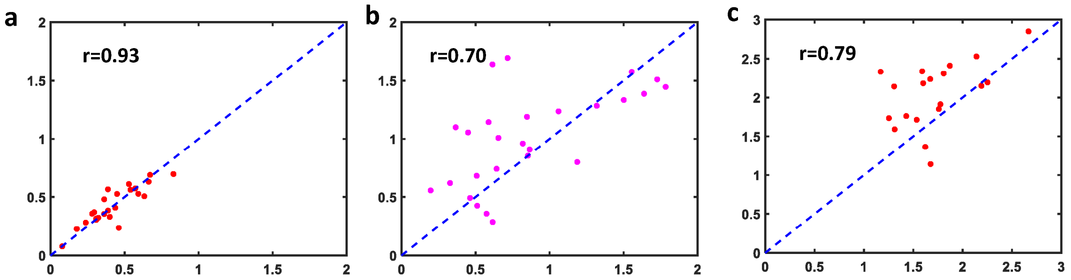

Supplementary Fig. 10 The correlation between predicted and measured fluxes. **a-c**, On the training set (**a**) and testing set (**b**), as well as for the alcohol dehydrogenase on the training set (**c**). Source data are provided as a Source Data file.

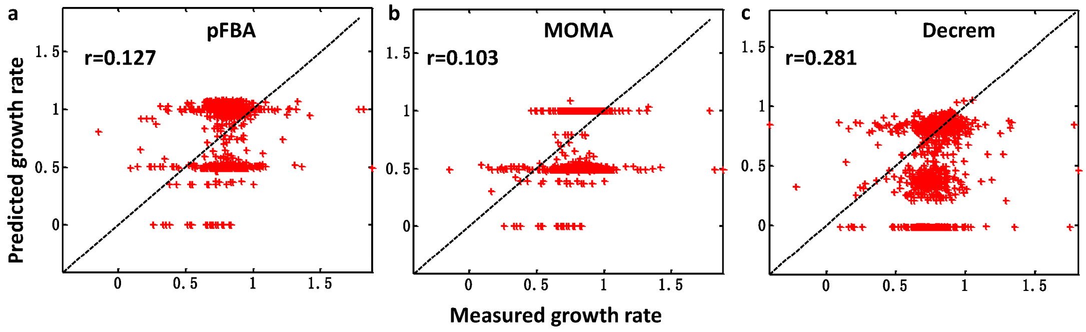

Supplementary Fig. 11 The growth rate distribution of the 1,030 metabolic genes knockout strains. a-c, The growth rates are predicted by the conventional methods: pFBA (**a**), MOMA (**b**), and Decrem (**c**), respectively, without any external flux constraints. Source data are provided as a Source Data file.

Supplementary Note 1: The data processing in the section of "Benchmark the metabolic fluxes prediction in response to environmental perturbation in vivo/vitro".

The genome-scale metabolic model of *E. coli* K12, *i*AF1260 [2] is used to test Decrem, because of its most complete metabolic network and abundant experimental metabolic data in various environments and gene-deletion mutants. The experimental data of intracellular fluxes are collected from the study by Gonzalez and colleagues [3]. Specifically, 124 reaction fluxes for *E. coli* BW25113 in MOPS medium (a K12 sub-strain [4]) were measured using ^{13}C metabolic flux analysis under four conditions: (1) glucose and aerobic respiration, (2) glucose and anaerobic respiration, (3) xylose and aerobic respiration, and (4) xylose and anaerobic respiration, separately. Furthermore, the relative mass of each biomass composition is quantified. All these nutrients are re-interpreted as bound constraints of associated metabolic reactions to approximate the real growth conditions in the flux balance analysis, as detailed in Supplementary Table 2. For each strain, 80 isotope-labeled intercellular metabolic fluxes matching more than 120 metabolic reactions in the *iAF1260* model, measured by ^{13}C MFA, are used to evaluate the fluxes predicted by all the methods used. The detailed metabolic fluxes and their corresponding reactions in the *iAF1260* are described in Supplementary Table 3.

In addition, the Yeast metabolic model, *i*MM904, and its 84 ^{13}C experiment fluxes of the wildtype S288C strains are utilized to evaluate the flux balance analysis methods [5]. The *i*YO840 metabolic model of *Bacillus subtilis* wildtype 168CA strain is used to fit the 32 ^{13}C experimental fluxes [6] (Supplementary Table 3). Similar to the above *E. coli* model, all these nutrients are re-interpreted as bound constraints of associated metabolic reactions to approximate the real growth conditions in the flux balance analysis (Supplementary Table 2).

For yeast mitochondrial compartment analysis, we first built a reference flux distribution for the *i*MM904 model using yeast wildtype ^{13}C isotope flux-constrained FBA and carried out metabolic flux prediction using Decrem and the original *i*MM904 model without ^{13}C-MFA constraint. Then, each predicted flux distribution is used to compare with the reference flux distribution to identify the intersection of nonzero flux reactions.

Two ^{13}C-MFA fluxes datasets with 28 mutant strains for *E. coli* and 38 mutant strains for Yeast are utilized to measure the predicted flux range [6-7]; that is, we predict flux variability range by FVA for each strain using Decrem and original GSM and then measure the Jaccard Index coefficient between ^{13}C-MFA fluxes and each prediction, the Jaccard Index is defined as:

$$J(\mathrm{A},\mathrm{B}) = \frac{\mathrm{A}\cap\mathrm{B}}{\mathrm{A}\cup\mathrm{B}} ,$$

where A and B represent the flux value interval of predicted and ^{13}C fluxes.

Supplementary Note 2: The data processing in the section of "Decrem accurately identifies the mutant fluctuation in yeast knockout strains".

The 38 mutants of Yeast from the knockout experiment of the pentose phosphate pathway (PPP), tricarboxylic acid (TCA) cycle, glyoxylate cycle, polysaccharide synthesis, mitochondrial transporters, and by-product formation [7], separately, are used to evaluate the performance of Decrem. Glucose is the sole carbon source for these 38 mutants. Here, we focused on the 38 genes from the central metabolism of the Yeast metabolic network that encode 28 flexible reactions that can catalyze a flux of at least 0.1% of the glucose uptake rate. The experimental ^{13}C fluxes, growth rate, external exchange fluxes and nutrient composition for each of the 38 strains are collected. Of these, the growth rate is used for method evaluation, and nutrients are used for the additional constraint of the metabolic model,

as detailed in Supplementary Table 4.

Supplementary Note 3: The workflow and data processing in the section of "Integrating global transcriptional regulation-derived key reaction kinetics into Decrem".

The multi-omics data of 24 mutant strains of *E. coli*, i.e., 85 gene expression, more than 100 metabolite concentrations, and 51 ^{13}C MFA metabolic fluxes, are collected from the experimental data by Ishii et. Al [1] (Supplementary Table 6). The metabolite-TF interactions are manually collected from the literature [8, 9, 10, 11], to explore the regulation relationship between metabolites and gene expression. The collections are then filtered by both their topological properties, i.e., hub nodes and branch points in the KEGG pathway [12], and the available metabolite concentration, leaving 45 metabolite candidates for further analysis. Two primary groups are generated by using a hierarchical cluster method on 45 metabolite concentrations, as shown in Supplementary Table 6: one with the metabolites mainly for the biomass compositions, i.e., the nucleic acids and amino acids, and the other mainly composing of the energy molecules and precursors, i.e., AMP, pyruvate, cAMP, et.al. Based on the regulatory interaction between metabolites and transcription factors generated from the experiment by Kochanowski et.al [11], we note that the biomass and precursor group could be regarded as primary global and local regulators for gene expression, respectively.

One statistics regression method, partial least squares regression (PLSR) (see Methods), is used to explore the possible quantitative linear regulatory relationship between the above metabolites and genes, based on the developed transcriptional regulation mechanism, with the metabolite concentration and gene expression level for the predictor and response variable, respectively. To void the false positives, the regression correlations are filtered with the strict cutoff: the total correlation should be greater than 0.84, and the first principal component should explain at least 38% of the observed gene expression. In the end, 32 of 85 genes are directly regulated by 23 biomass metabolites, as shown in Supplementary Fig 8. To validate the significance of the result, we first calculated the canonical correlation between the identified genes and 23 biomass metabolites on Ishii et al. and our experimental dataset (Fig 5c and Supplementary Fig 7c) to validate the correlation consistency. Then, randomly selected 10,000 combinations of metabolites (with a number in the range of 15-25) from the above 45 metabolites, and fitted the above 32 genes by the concentration for each combination, as shown in Fig. 5e. This benchmark analysis demonstrates that the total correlation calculated by our predicted metabolites-gene interactions are significantly higher than the predictions from those randomly selected combinations (p-value = 3.1E-3), while the precursor groups are not significant (p-value = 0.48). The most important is that most of the identified BG metabolites are validated by a recent study of metabolite concentration prediction derived from the quantitative proteome [13]. Interestingly, most of these 32 genes are mainly from the alternative PPP and reactions related to major metabolic precursors/secretions within pyruvate metabolism (i.e., pyruvate, ethanol, and acetate), instead of glycolysis and TCA cycle, as shown in Fig. 6a, which agrees with the fact that the synthesis in PPP, i.e., NADPH and the ribose 5-phosphate, and the essential pyruvate metabolism are related to the growth [14].

Then, we reconstructed a kinetic model integrated with regulation for those growth-associated metabolic reactions. Specifically, we combined the reactant metabolites (regulator metabolites) and BP-represented gene (enzyme) expression to reformulate a linear Michaelis-Menten equation (see Methods). This kinetic regulatory model depended only on the concentrations of associated metabolites and was supported by the following two sources of information. First, the biomass/growth

rate-derived global regulation and substrate metabolites, rather than the dynamic local regulator metabolites, play a key role in the flux distribution in our selected reactions, which is also observed previously [15, 16], as those reactions often respond to environmental perturbations with few global TF regulators, such as Crp-cAMP, phosphotransferase system et al. Second, the concentrations of primary carbon metabolites in central metabolism were at levels approximate to or higher than their Michaelis constants: K_m [17], which allows derivation of linearized approximation of Michaelis-Menten kinetics (see Methods). Based on these two lines of prior knowledge, we used a linear regression method to fit identified cell state-associated reactions and quantify the corresponding parameters according to the empirical ^{13}C FMA fluxes and metabolite concentrations (see Methods).

Based on gene expression predicted by biomass metabolites, we identify global regulated reactions that their flux coordination supported by the biological well-known global regulation mechanism [16, 18, 19], and available substrate concentrations (Supplementary Fig 9 and Fig 6b). We built the complete kinetic model for five key regulation reactions in central metabolism, i.e., Hexokinase, Acetate kinase, Pyruvate dehydrogenase, Fumarate reductase, and Alcohol dehydrogenase, and the correlation of predicted fluxes with the experiments reached to 0.95, 0.98, 0.93, 0.93, and 0.79 respectively. To validate the linear kinetic of the identified five reactions, we test them on a multi-omics dataset with 45-time points and also achieve a good performance. The regression coefficient of those reactions validate that the BG metabolites play a dominant role in those global-regulated flux prediction (Supplementary Table 7, Fig. 6c and Supplementary Fig. 10). Based on the result above, the built kinetic model can be applied to other strains to predict the regulation fluxes based on their specific of substrate concentration.

Supplementary Note 4: The workflow and data processing in the section of "Growth rate estimation for E. coli genome-scale gene deletion strains using Decrem integrated with global regulation kinetics".

3,800 single-gene knockout experimental strains [20] are filtered based on the *E. coli iAF1260* metabolic model. Specifically, by mapping 3,800 knockout genes into the metabolic gene list of the *iAF1260* model, we note that only 1,030 genes can be mapped to 851 enzymes corresponding to 1324 reactions [20]. So, 1,030 strains are kept for further analysis (Supplementary Table 8). The metabolites of 1,030 strains are normalized by pooling their concentrations together and then re-scaling them using the corresponding metabolite concentrations in the wildtype strain of Supplementary Material 3 as reference. In addition, the nutrient compositions are regarded as the constraints of the corresponding exchange reactions of the *iAF1260* model when conducting the kinetic and non-kinetic methods (Supplementary Table 8). We then separately construct essential regulation kinetic fluxes based on the following metabolite and substrate concentrations, namely the Hexokinase, Acetate kinase, Pyruvate dehydrogenase, Fumarate reductase, and Alcohol dehydrogenase (see Methods, Fig. 6a and Supplementary Table 8), according to the rebuilt Michaels kinetic model in the corresponding main context. Then, we construct the kinetic Decrem and other compared methods by shrinking the prediction of 5 regulation fluxes to their kinetic estimations (see the corresponding main context).

After performing the kinetic Decrem analysis for each strain, the total flux for each pathway is calculated by cumulative summation of predicted fluxes within the same pathway. The knowledge-based pathways related to *E. coli* growth are manually collected from literature [7, 8, 12], and the Spearman and Pearson correlation between each of them and the experimental growth rate are used to measure its interpretability for the growth rate, as detailed in Supplementary Table 8. Then, for each pathway,

we accumulate the predicted growth rates over all strains, and then calculate the correlation between this accumulation and experimental growth rate for each comparison method, as shown in Supplementary Table 8.

To analyze the specific influence of Decrem on topology, we perform Principal Component Analysis (PCA) to the predicted flux over all 1,030 strains. Then, the largest two principal components (PCs) are used to explain the variance of these predicted fluxes, and then PCs were classified into the sparse linear basis group and the regular reaction group. In addition, the effect of the sparse linear basis on single gene knockout is examined by comparing the mean and the distribution of experimental growth rate for the mutant strains between the following three groups: the regular reaction group, the sparse linear basis consisting of only one component reaction, and the sparse linear basis containing multiple component reactions. Finally, each sparse linear basis is classified based on the property of its catalytic enzyme (e.g., monomeric and multimeric) and the number of simple cycles in them.

Supplementary Note 5: **The Decrem algorithm.**

Section 1: The exchange reactions are added to the dense reaction cycle subnetwork:

One given dense reaction cycle subnetwork $\mathbf{C}_k$ can be represented as a stoichiometric matrix $\mathbf{S}_{C_k}$, with rows for metabolites and columns for reactions. The uptake and secreted metabolites for each subnetwork can be identified by scanning the rows of its stoichiometric matrix. If all the elements for each row are non-positive values, then the corresponding metabolite for this row is regarded as an uptake metabolite. Conversely, if all the elements for each row are non-negative values, then the corresponding metabolite for this row is regarded as a secreted metabolite. For each of the identified uptake metabolites or secreted metabolites, an additional exchange reaction is added to the stoichiometric matrix $\mathbf{S}_{C_k}$ by adding one identity column vector filled with -1 or 1 at the position indexed by this metabolite into $\mathbf{S}_{C_k}$, to avoid the accumulation of the uptake or secreted metabolites in subnetwork $\mathbf{C}_k$, respectively.

Section 2: The procedure to derive sparse linear basis vectors:

Based on the definition of MMP, the shortest and linear independent pathway set $\mathbf{N}_{C_k}^S$, for a stoichiometric matrix $\mathbf{S}_{C_k}$ of a subnetwork $\mathbf{C}_k$, with dimensions $m_{C_k} \times n_{C_k}$, can be determined as:

$$\mathrm{MMP}(\mathbf{S}_{C_k}) = \mathbf{N}_{C_k}^S = \arg\min_{N_{C_k}} \left\|\mathbf{N}_{C_k}\right\|_0$$

$$s.t.\, col(\mathbf{N}_{C_k}) = null(\mathbf{S}_{C_k})$$

where $null(\mathbf{S}_{C_k})$ is the null space of $\mathbf{S}_{C_k}$, and marked as $\mathbf{N}_{C_k}$, which is equal to the following algorithm:

A greedy algorithm for sparse null space problem:

Initialize: stoichiometric matrix $\mathbf{S}_{C_k}$ and $\mathbf{N}_{C_k}^{S_m} = \emptyset, \mathbf{N}_{C_k}^S = \emptyset$.

For $m = 1, \ldots, r_k$

Find the sparsest null vector $\mathbf{v}_{C_k}^m$ such that rank $(\mathbf{N}_{C_k}^{S_m} \oplus \mathbf{v}_{C_k}^m) = m$

$\mathbf{N}_{C_k}^{S_m} = \mathbf{N}_{C_k}^{S_m} \oplus \mathbf{v}_{C_k}^m$

End

$\mathbf{N}_{C_k}^{S} = \mathbf{N}_{C_k}^{S_m}$

where $r_k = l_k - rank(\mathbf{S}_{C_k})$ and l_k is the number of $\mathbf{S}_{C_k}$.

Bian and his colleagues have proved that a matrix $\mathbf{N}_{C_k}^{S}$ is the sparsest null basis of $\mathbf{S}_{C_k}$ if and only if it can be constructed by the greedy algorithm [21]. However, finding the sparsest null vectors is an NP-hard problem. We can solve the corresponding $\mathrm{L1\ norm}$ relaxation by using the following formulation, and then each of $\mathbf{v}_{C_k}^{m}$ can be represented as:

$$\mathbf{v}_{C_k}^{m} = arg \min_{\mathbf{v}_{C_k}^{m}} \left\| \mathbf{v}_{C_k}^{m} \right\|_1$$

$$s.t.\ \mathbf{S}_{C_k}.\mathbf{v}_{C_k}^{m} = 0$$

$$\mathbf{P}_{C_k}^{N_m}.\mathbf{v}_{C_k}^{m} \neq 0$$

where $\mathbf{P}_{C_k}^{N_m}$ represents the projection matrix onto the null space $\mathbf{N}_{C_k}^{S_m}$ as the mth iteration; the condition $\mathbf{P}_{C_k}^{N_m}.\mathbf{v}_{C_k}^{m} \neq 0$ implies that $\mathbf{v}_{C_k}^{m}$ is not in the current span of $\mathbf{N}_{C_k}^{S_m}$, and hence rank $(\mathbf{N}_{C_k}^{S_m} \oplus \mathbf{v}_{C_k}^{m}) = m$. This procedure is detailed by the linear programming problem in the step 2 section of "Reconstruction of GSMs with topologically decoupled reactions" in the main text and Supplementary Algorithm 1.

Algorithm 1: Solving sparse basis for reactions cluster $\mathbf{C}_k$
Inputs: $\mathbf{S}_{C_k}$ with $r_k = l_k - rank(\mathbf{S}_{C_k})$, the stoichiometric matrix of the reactions cluster $\mathbf{C}_k$, lower and upper bounds (lb, ub).
Outputs: feasible sparse basis matrix $\mathbf{N}_{C_k}^{S}$.

Set $m \leftarrow 0, \mathbf{P}_{C_k}^{N_m} \leftarrow 0, \mathbf{N}_{C_k}^{S_m} \leftarrow 0$

$w \leftarrow$ random weights.
While $m < r_k$

$\mathbf{P}_{C_k}^{N_m} \leftarrow \mathbf{I} - \mathbf{P}_{C_k}^{N_m}$

$\mathbf{v}_{C_k}^{m+}$
$\leftarrow$ optimization of the linear programming problem in step 2 of the section "Reconstruction of GSMs with topologically decoupled reactions" with constraint $\mathbf{w}^T.\mathbf{P}_{C_k}^{N_m}.\mathbf{v}_{C_k}^{m} > \zeta$

$\mathbf{v}_{C_k}^{m-}$
$\leftarrow$ optimization of the linear programming problem in step 2 of the section "Reconstruction of GSMs with topologically decoupled reactions" with constraint $\mathbf{w}^T.\mathbf{P}_{C_k}^{N_m}.\mathbf{v}_{C_k}^{m} < -\zeta$

Set $\mathbf{v}_{C_k}^{m} \leftarrow argmin\left(\left\|\mathbf{v}_{C_k}^{m+}\right\|_0, \left\|\mathbf{v}_{C_k}^{m-}\right\|_0\right)$

If empty $(\mathbf{v}_{C_k}^{m})$ then
Break

Else

$$\mathbf{N}_{C_k}^{S_{m+1}} \leftarrow \mathbf{N}_{C_k}^{S_m} \oplus \mathbf{v}_{C_k}^{m}$$

$$\widehat{\mathbf{N}}_{C_k}^{m+1} \leftarrow \text{OrthogonalBasisUsingSVD}\left(\mathbf{N}_{C_k}^{S_{m+1}}\right)$$

$$\mathbf{P}_{C_k}^{N_{m+1}} \leftarrow \widehat{\mathbf{N}}_{C_k}^{m+1}.\widehat{\mathbf{N}}_{C_k}^{m+1^T}$$

End if

Set $m = m + 1$

End while

$$\mathbf{N}_{C_k}^{S} = \mathbf{N}_{C_k}^{S_m}$$

Return $\mathbf{N}_{C_k}^{S}$

Supplementary Note 6: Parsimonious Decrem.

Step1: We optimize the predefined objective function (reactions) by Decrem, as the procedure of step 3 in the section "Reconstruction of GSMs with topologically decoupled reactions" in the main context:

$$\max_{\mathbf{v}^{IR}} \mathbf{c}\mathbf{v}^{IR}$$

$$\text{s.t.} \quad \mathbf{S}^{IR}.\mathbf{v}^{IR} = 0$$

$$\mathbf{S}^{IR} = \left[\mathbf{S}^{NC}, \mathbf{S}_{C_1}^{IPR}, \dots, \mathbf{S}_{C_K}^{IPR}\right] = \left[\mathbf{S}^{NC}, \mathbf{S}_{C_1}^{*}\mathbf{N}_{C_1}^{S^*}, \dots, \mathbf{S}_{C_K}^{*}\mathbf{N}_{C_K}^{S^*}\right]$$

$$\mathbf{v}^{IR} = \left[\mathbf{v}^{NC}, \mathbf{v}_{C_1}^{IPR}, \dots, \mathbf{v}_{C_K}^{IPR}\right]^T$$

$$\mathbf{v}_{C_k}^{IPR} = \left[\mathbf{v}_{C_k}^{1}, \dots, \mathbf{v}_{C_k}^{r_k}\right]^T$$

$$\mathbf{lb}^{NC} \leq \mathbf{v}^{NC} \leq \mathbf{ub}^{NC}$$

$$\max\left(f\left(lb_{C_k}^{i}, NZ\left(\mathbf{N}_{C_k}^{i}\right)\right)./f\left(\mathbf{N}_{C_k}^{i}, NZ\left(\mathbf{N}_{C_k}^{i}\right)\right)\right)$$

$$\leq v_{C_k}^{i} \leq$$

$$\min\left(f\left(ub_{C_k}^{i}, NZ\left(\mathbf{N}_{C_k}^{i}\right)\right)./f\left(\mathbf{N}_{C_k}^{i}, NZ\left(\mathbf{N}_{C_k}^{i}\right)\right)\right)$$

$$i = 1, \dots, r_k; k = 1, \dots, K$$

Step 2: The Parsimonious Decrem can be represented as:

$$\min_{v^{IR}} \left|\left|\mathbf{v}^{IR}\right|\right|_{L1}$$

$$\text{s.t.} \quad \mathbf{S}^{IR}.\mathbf{v}^{IR} = 0$$

$$\mathbf{v}^{obj} = \max \mathbf{c}\mathbf{v}^{IR}$$

This indicates the minimal L1 norm of all the predicted fluxes with the constraints of the maximal objective fluxes from step 1.

Supplementary Note 7: Deriving the approximate linear kinetic model for global regulated reactions.

Given

$$-\log\big(\mathrm{v}([S],[P],[A],[I],[E])\big) = \underbrace{-\log([E])}_{\text{Enzyme term}} \underbrace{-\log\big(1-[P]/[S]\,e^{-\Delta_r G'^{\circ}/RT}\big) + \sum_v \log(1+[I_v])}_{\text{Metabolite associated}}$$

$$\underbrace{-\log(k^+) - \sum_v k_I^v}_{\text{Kinetic constants}} + \sum_u \log\left(1+\frac{k_A^u}{[A_u]}\right) + \log\left(1+\frac{k_m^s}{[S]}\left(1+\frac{[P]}{k_m^p}\right)\right)$$

Kinetic constants Nonlinear terms

And

$$\mathrm{Log}\big(E_g\big) \approx \alpha_g \textstyle\sum_{j=1}^{N} \theta_j \log\big(M_{bj}\big) + \sum_{i=1}^{K} \beta_{gi} \log\big(M_{gi}\big) + b$$

where M_{bj} indicates the biomass metabolites and M_{gi} represents the TF regulating metabolites. We reexamined the nonlinear terms of the above equation based on the knowledge that systemic experimental analysis revealed that $[S]$ was $\geq k_m^s$ for almost all of the metabolites in the central metabolism of three model organisms [17]; hence, we have $\frac{k_m^s}{[S]} \leq 1$.

Situation 1: For the growth state-regulated irreversible reaction, if we have $\frac{[P]}{k_m^p} \to 0$, and $\frac{k_A^u}{[A_u]} \leq 1$, then the Taylor expansion of the above equation is as follows:

$$-\log\big(\mathrm{v}([S],[P],[A],[I],[E])\big)$$

$$= -\alpha_g \sum_{j=1}^{N} \theta_j \log\big(M_{bj}\big) - \sum_{i=1}^{K} \beta_{gi} \log\big(M_{gi}\big) - b$$

$$-\log\big(1-[P]/[S]\,e^{-\Delta_r G'^{\circ}/RT}\big) + \sum_v \log(1+[I_v])$$

$$-\log(k^+) - \sum_v k_I^v + \frac{k_m^s}{[S]} + \sum_u \left(\frac{k_A^u}{[A_u]}\right) + O\left(\frac{k_m^s}{[S]}, \frac{k_A^u}{[A_u]}\right)$$

Situation 2: For the generalized reversible reaction in which the enzyme is substrate or product saturated (or both): $\frac{k_m^s}{[S]} \leq 1$ and $\frac{k_m^p}{[P]} \leq 1$. Assuming $v > 0$, we have the following:

$$-\log(k^+) + \log\left(1+\frac{k_m^s}{[S]}\left(1+\frac{[P]}{k_m^p}\right)\right) \approx \log\left(\frac{1}{k^+} + \frac{1}{k^+}\frac{[P]}{[S]}\frac{k_m^s}{k_m^p}\right) = \log\left(\frac{1}{k^+} + \frac{[P]/[S]}{k^{eq}}\frac{1}{k^-}\right)$$

Here, $[P]/[S] < k^{eq}$ according to the reaction Gibbs energy, $\frac{1}{k^+} \leq 1$ and $\frac{1}{k^-} \leq 1$ for the significantly regulated reactions, and $\frac{1}{k^+} + \frac{[P]/[S]}{k^{eq}} 1k— 1 < 1$; so, the Taylor expansion of above is as follows:

$$\log\left(\frac{1}{k^+} + \frac{[P]/[S]}{k^{eq}}\frac{1}{k^-}\right) = \log\left(1 + \frac{1}{k^+} + \frac{[P]/[S]}{k^{eq}}\frac{1}{k^-} - 1\right)$$

$$= \frac{1}{k^+} + \frac{[P]/[S]}{k^{eq}}\frac{1}{k^-} - 1 + O(\frac{1}{k^+} + \frac{[P]/[S]}{k^{eq}}\frac{1}{k^-})$$

$$- \log\big(\mathrm{v}([S], [P], [A], [I], [E])\big)$$

$$\approx -\alpha_g \sum_{j=1}^{N} \theta_j \log(M_{bj}) - \sum\nolimits_{i=1}^{K} \beta_{gi} \log(M_{gi}) - b - \log\left(1 - [P]/[S]\, e^{-\Delta_r G'^{\circ}/RT}\right)$$

$$+ \sum_v \log(1 + [I_v])$$

$$- \sum_v k_I^v + \sum_u \left(\frac{k_A^u}{[A_u]}\right) + \frac{1}{k^+} + \frac{[P]/[S]}{k^{eq}}\frac{1}{k^-} - 1$$

where all the bold variables in equations are kinetic parameters. Similarly, when $v > 0$, we have $-\log(k^-) + \log\left(1 + \frac{k_m^p}{[P]}\left(1 + \frac{[S]}{k_m^s}\right)\right) \approx \log\left(\frac{1}{k^-} + \frac{[S]/[P]}{k^{eq}}\frac{1}{k^+}\right)$.

Togethering the above two situations, we can reformulate them as only metabolite-based linear kinetic:

$$\log\big(\mathrm{v}([S], [P], [A], [I], [E])\big)$$

$$\approx \alpha_g \sum_{j=1}^{N} \theta_j \log(M_{bj}) + \sum\nolimits_{i=1}^{K} \beta_{gi} \log(M_{gi}) + \log\left(1 - [P]/[S]\, e^{-\Delta_r G'^{\circ}/RT}\right)$$

$$+ \frac{[P]/[S]}{k^{eq}} + \sum_u \left(\frac{k_A^u}{[A_u]}\right) + constant$$

Considering the globally regulated genes can be simplified as $\log(E_g) \approx \alpha_g \sum_{j=1}^{N} \theta_j \log(M_{bj})$ through the section of "Gene expression estimation", we finally have:

$$\log\big(\mathrm{v}([S], [P], [A], [I], [E])\big)$$

$$\approx \alpha_g \sum_{j=1}^{N} \theta_j \log(M_{bj}) + \log\left(1 - [P]/[S]\, e^{-\Delta_r G'^{\circ}/RT}\right) + \frac{[P]/[S]}{k^{eq}} + \sum_u \left(\frac{k_A^u}{[A_u]}\right)$$

$$+ constant$$

Supplementary References

1. Ishii N*, et al.* Multiple high-throughput analyses monitor the response of E. coli to perturbations. *Science* **316**, 593-597 (2007).

2. Feist AM*, et al.* A genome-scale metabolic reconstruction for Escherichia coli K-12 MG1655 that accounts for 1260 ORFs and thermodynamic information. *Mol Syst Biol* **3**, 121 (2007).

3. Gonzalez JE, Long CP, Antoniewicz MR. Comprehensive analysis of glucose and xylose metabolism in Escherichia coli under aerobic and anaerobic conditions by (13)C metabolic flux analysis. *Metab Eng* **39**, 9-18 (2017).

4. Baba T*, et al.* Construction of Escherichia coli K-12 in-frame, single-gene knockout mutants: the Keio collection. *Mol Syst Biol* **2**, 2006 0008 (2006).

5. Moxley JF*, et al.* Linking high-resolution metabolic flux phenotypes and transcriptional regulation in yeast modulated by the global regulator Gcn4p. *Proc Natl Acad Sci U S A* **106**, 6477-6482 (2009).

6. Ruhl M, Le Coq D, Aymerich S, Sauer U. 13C-flux analysis reveals NADPH-balancing transhydrogenation cycles in stationary phase of nitrogen-starving Bacillus subtilis. *J Biol Chem* **287**, 27959-27970 (2012).

7. Blank LM, Kuepfer L, Sauer U. Large-scale 13C-flux analysis reveals mechanistic principles of metabolic network robustness to null mutations in yeast. *Genome Biol* **6**, R49 (2005).

8. Keseler IM*, et al.* The EcoCyc database: reflecting new knowledge about Escherichia coli K-12. *Nucleic Acids Res* **45**, D543-D550 (2017).

9. Schomburg I, Jeske L, Ulbrich M, Placzek S, Chang A, Schomburg D. The BRENDA enzyme information system-From a database to an expert system. *J Biotechnol* **261**, 194-206 (2017).

10. Placzek S*, et al.* BRENDA in 2017: new perspectives and new tools in BRENDA. *Nucleic Acids Res* **45**, D380-D388 (2017).

11. Kochanowski K, Gerosa L, Brunner SF, Christodoulou D, Nikolaev YV, Sauer U. Few regulatory metabolites coordinate expression of central metabolic genes in Escherichia coli. *Mol Syst Biol* **13**, 903 (2017).

12. Kanehisa M, Sato Y, Kawashima M, Furumichi M, Tanabe M. KEGG as a reference resource for gene and protein annotation. *Nucleic Acids Res* **44**, D457-462 (2016).

13. Zelezniak A*, et al.* Machine Learning Predicts the Yeast Metabolome from the Quantitative

Proteome of Kinase Knockouts. *Cell Syst* **7**, 269-283 e266 (2018).

14. Flamholz A, Noor E, Bar-Even A, Liebermeister W, Milo R. Glycolytic strategy as a tradeoff between energy yield and protein cost. *Proc Natl Acad Sci U S A* **110**, 10039-10044 (2013).

15. Uematsu S, *et al.* Multi-omics-based label-free metabolic flux inference reveals obesity-associated dysregulatory mechanisms in liver glucose metabolism. *iScience* **25**, 103787 (2022).

16. Zampieri M, Horl M, Hotz F, Muller NF, Sauer U. Regulatory mechanisms underlying coordination of amino acid and glucose catabolism in Escherichia coli. *Nat Commun* **10**, 3354 (2019).

17. Park JO, *et al.* Metabolite concentrations, fluxes and free energies imply efficient enzyme usage. *Nat Chem Biol* **12**, 482-489 (2016).

18. Ramon C, Gollub MG, Stelling J. Integrating -omics data into genome-scale metabolic network models: principles and challenges. *Essays Biochem* **62**, 563-574 (2018).

19. Kochanowski K, Okano H, Patsalo V, Williamson J, Sauer U, Hwa T. Global coordination of metabolic pathways in Escherichia coli by active and passive regulation. *Mol Syst Biol* **17**, e10064 (2021).

20. Fuhrer T, Zampieri M, Sevin DC, Sauer U, Zamboni N. Genomewide landscape of gene-metabolome associations in Escherichia coli. *Mol Syst Biol* **13**, 907 (2017).

21. Bian X, Krim H, Bronstein A, Dai L. Sparse null space basis pursuit and analysis dictionary learning for high-dimensional data analysis. In: *2015 IEEE International Conference on Acoustics, Speech and Signal Processing (ICASSP)*. IEEE (2015).
