# Supplementary material for: Local flux coordination and global gene expression regulation in metabolic modeling
Source: Nat Commun. 2023 Sep 14;14:5700. doi: 10.1038/s41467-023-41392-6 (PMC10502109; doi:10.1038/s41467-023-41392-6)
Supplement: Supplementary file 2 — Reporting Summary [file 41467_2023_41392_MOESM2_ESM.pdf]

nature portfolio

Corresponding author(s): Wei Du, Huansheng Cao

Last updated by author(s): Aug 25, 2023

# Reporting Summary

Nature Portfolio wishes to improve the reproducibility of the work that we publish. This form provides structure for consistency and transparency in reporting. For further information on Nature Portfolio policies, see our Editorial Policies and the Editorial Policy Checklist.

## Statistics

For all statistical analyses, confirm that the following items are present in the figure legend, table legend, main text, or Methods section.

| n/a | Confirmed | |
|---|---|---|
| ☐ | ☒ | The exact sample size (*n*) for each experimental group/condition, given as a discrete number and unit of measurement |
| ☐ | ☒ | A statement on whether measurements were taken from distinct samples or whether the same sample was measured repeatedly |
| ☐ | ☒ | The statistical test(s) used AND whether they are one- or two-sided<br>*Only common tests should be described solely by name; describe more complex techniques in the Methods section.* |
| ☒ | ☐ | A description of all covariates tested |
| ☒ | ☐ | A description of any assumptions or corrections, such as tests of normality and adjustment for multiple comparisons |
| ☐ | ☒ | A full description of the statistical parameters including central tendency (e.g. means) or other basic estimates (e.g. regression coefficient) AND variation (e.g. standard deviation) or associated estimates of uncertainty (e.g. confidence intervals) |
| ☐ | ☒ | For null hypothesis testing, the test statistic (e.g. *F*, *t*, *r*) with confidence intervals, effect sizes, degrees of freedom and *P* value noted<br>*Give P values as exact values whenever suitable.* |
| ☒ | ☐ | For Bayesian analysis, information on the choice of priors and Markov chain Monte Carlo settings |
| ☒ | ☐ | For hierarchical and complex designs, identification of the appropriate level for tests and full reporting of outcomes |
| ☐ | ☒ | Estimates of effect sizes (e.g. Cohen's *d*, Pearson's *r*), indicating how they were calculated |

*Our web collection on statistics for biologists contains articles on many of the points above.*

## Software and code

Policy information about availability of computer code

| | |
|---|---|
| Data collection | no software are used |
| Data analysis | Decrem is implemented as a Matlab R2020a package. The source code, user tutorial and demo are available at GitHub (https://github.com/lgyzngc/Decrem-1.0.git) and Zenodo (https://doi.org/10.5281/zenodo.8275285). We also compared the performance of our Decrem with other five methods that are utilized to flux prediction and analysis: FBA, pFBA, FVA, REPPS and RELATCH, The cobra 2.0.5 package is utilized to implement the FBA, pFBA and FVA analysis, REPPS package is download on the address:https://academic.oup.com/bioinformatics/article/33/6/893/2725488?searchresult=1#supplementary-data, and the RELATCH can be found in https://genomebiology.biomedcentral.com/articles/10.1186/gb-2012-13-9-r78#MOESM12 (Additional File 12: Implementation of RELATCH. RELATCH is implemented using the COBRA Toolbox for MATLAB. (ZIP 173 KB)). |

For manuscripts utilizing custom algorithms or software that are central to the research but not yet described in published literature, software must be made available to editors and reviewers. We strongly encourage code deposition in a community repository (e.g. GitHub). See the Nature Portfolio guidelines for submitting code & software for further information.

# Data

Policy information about availability of data

All manuscripts must include a data availability statement. This statement should provide the following information, where applicable:

- Accession codes, unique identifiers, or web links for publicly available datasets
- A description of any restrictions on data availability
- For clinical datasets or third party data, please ensure that the statement adheres to our policy

All data used are publicly available. The original and reconstructed metabolic models are available online: original metabolic models are available at BIGG models http://bigg.ucsd.edu/models/iND750, http://bigg.ucsd.edu/models/iMM904 , http://bigg.ucsd.edu/models/iML1515, http://bigg.ucsd.edu/models/iAF1260 and reconstructed metabolic models of the four reconstructed models, iAF1260, iML1515, iMM904, and iDN750, are available at https://github.com/lgyzngc/Decrem-1.0/tree/master/three%20reconstructed%20models. All used exchange reactions, nutrient uptake, experimental growth rates, 13C fluxes and gene expression for Decrem modeling and metabolic simulation are found in Supplementary Data. And the LS-MS data is sourced from https://www.ebi.ac.uk/biostudies for genome-scale mutant strains of E. coli. The RNAseq data generated in this study have been deposited in the NCBI SRA(https://www.ncbi.nlm.nih.gov/sra) database under accession code PRJNA910919 (https://www.ncbi.nlm.nih.gov/bioproject/?term=PRJNA910919). The metabolome data are available at Metabolomics Workbench with trackID 3535 (https://www.metabolomicsworkbench.org/data/DRCCDataDeposit.php?Mode=SetupListDataUpload&UploadMode=ListDataUpload). and the LS-MS data are available with a EBI web link of www.ebi.ac.uk/metabolights/MTBLS8467. ALL data acquired in this study are available in a public Zenodo repository (https://doi.org/10.5281/zenodo.8285102)

# Research involving human participants, their data, or biological material

Policy information about studies with human participants or human data. See also policy information about sex, gender (identity/presentation), and sexual orientation and race, ethnicity and racism.

| | |
|---|---|
| Reporting on sex and gender | N/A |
| Reporting on race, ethnicity, or other socially relevant groupings | N/A |
| Population characteristics | N/A |
| Recruitment | N/A |
| Ethics oversight | N/A |

Note that full information on the approval of the study protocol must also be provided in the manuscript.

# Field-specific reporting

Please select the one below that is the best fit for your research. If you are not sure, read the appropriate sections before making your selection.

[x] Life sciences [ ] Behavioural & social sciences [ ] Ecological, evolutionary & environmental sciences

For a reference copy of the document with all sections, see nature.com/documents/nr-reporting-summary-flat.pdf

# Life sciences study design

All studies must disclose on these points even when the disclosure is negative.

| | |
|---|---|
| Sample size | Strain and culturing. E. coli strain BW25113 was grown in MOPS minimal medium (Teknova Inc, California, USA) with glucose at 2g/L with shaking at 120 rpm at 37°C. Aliquots of cells were collected at four growth states (timepoints): the beginning of the lag phase, the transition from lag to log phase, the mid-log phase, and the early stationary phase. In total, 12 samples are included in this study, three replicates per growth condition and time point. No statistic methods used to predetermine sample size, no no sample size calculation was performed, the sample was choosing by the growth state of E. coli. |
| Data exclusions | no data excluded |
| Replication | all attempts at replication were successful |
| Randomization | randomization of the samples were performed in terms of positions in shaker and timing of sampling |
| Blinding | samples need to be sampled at different timepoints, and therefor can not be blinded |

# Reporting for specific materials, systems and methods

We require information from authors about some types of materials, experimental systems and methods used in many studies. Here, indicate whether each material, system or method listed is relevant to your study. If you are not sure if a list item applies to your research, read the appropriate section before selecting a response.

## Materials & experimental systems

| n/a | Involved in the study | |
|---|---|---|
| ☒ | ☐ | Antibodies |
| ☒ | ☐ | Eukaryotic cell lines |
| ☒ | ☐ | Palaeontology and archaeology |
| ☒ | ☐ | Animals and other organisms |
| ☒ | ☐ | Clinical data |
| ☒ | ☐ | Dual use research of concern |
| ☒ | ☐ | Plants |

## Methods

| n/a | Involved in the study | |
|---|---|---|
| ☒ | ☐ | ChIP-seq |
| ☒ | ☐ | Flow cytometry |
| ☒ | ☐ | MRI-based neuroimaging |
