# Supplementary material for: Local flux coordination and global gene expression regulation in metabolic modeling
Source: Nat Commun. 2023 Sep 14;14:5700. doi: 10.1038/s41467-023-41392-6 (PMC10502109; doi:10.1038/s41467-023-41392-6)
Supplement: Supplementary file 3 — Description of Additional Supplementary Files [file 41467_2023_41392_MOESM3_ESM.pdf]

Title: Supplementary Data 1

Description: The annotation of reconstruction decoupled metabolic models. The reconstructed models can be found in Data availability.

Title: Supplementary Data 2

Description: Nutrient uptake reactions for three model microorganisms under six growth conditions.

Title: Supplementary Data 3

Description: The 13C MFA fluxes for three model microorganisms under six growth conditions.

Title: Supplementary Data 4

Description: The dataset used to predict the fluxes and growth rates for the 38 Yeast deletion strains.

Title: Supplementary Data 5

Description: The correlation statistic between the experimental and predicted metabolic fluxes and growth rates for the 38 Yeast deletion strains.

Title: Supplementary Data 6

Description: The datasets used to build the global regulation model.

Title: Supplementary Data 7

Description: The datasets used to validate the global regulation model.

Title: Supplementary Data 8

Description: The datasets used to construct kinetic Decrem to predict the growth rates on genome-scale deletion strains.
